# Veterinary Medical Society of New York County

**Published:** 1896-12

**Authors:** Robert W. Ellis

**Affiliations:** Secretary


### VETERINARY MEDICAL SOCIETY OF NEW YORK COUNTY.

THE regular monthly meeting was called to order Wednesday, November 4, 1896, at 8.45 P.M., by the President, Dr. Huidekoper, at the Academy of Medicine. On roll-call the following members responded, viz.: Drs. Delaney, Ellis, Foy, Giffen, Gill, Huidekoper, Hanson, Jackson, Lamkin, MacKellar, Neher, O'Shea, and Robertson. The minutes of the previous meeting were read, and, after slight correction, approved.

Dr. Gill, Chairman of the Board of Censors, reported favorably on the names of the gentlemen who had applied for membership, but, owing to a slight error in the form of applications, the chair directed that the names be withdrawn for action until the application be presented in proper form. The board also recommended to the Society that the Secretary be authorized to notify all members eighteen months in arrears that if said arrears were not settled by the next meeting their names would be dropped from the roll. Moved and seconded that the report be accepted; carried.

Dr. Gill reported a case of "Osteoporosis," which was followed by an interesting discussion, opened by Dr. Hanson and participated in by Drs. Neher, Robertson, and others.

Dr. Giffen next reported a case of "Hemorrhage from the Lungs in a Horse," with recovery, followed by a relapse ten days later, which resulted in death ten minutes from time of attack. This case was freely discussed by Drs. Robertson, Gill, Neher, and Hanson.

Dr. Robertson then reported a post-mortem which revealed a case of "Osteo-sarcoma," which he had not had the opportunity of seeing alive; but which had been diagnosed as a case of pharyngeal paralysis. A very interesting discussion by Drs. Gill, Hanson, Jackson, and others followed.

Dr. Gill next reported a case of "Prolapse of the Vagina in a Bitch," and Dr. Jackson a case of "Fracture of the Facial Bones," due to a runaway.

Dr. O'Shea, Chairman of the Judiciary Committee, reported that the committee had a warrant out for one illegal practitioner, to be served as soon as they can locate his present abode, and that they have other cases on which they are going to proceed immediately, and that in reference to the case of compelling a practitioner to remove an assumed degree from after his name the committee would consult the Association's counsel before proceeding. Moved and seconded that the report be accepted; carried.

Dr. Robertson, Chairman of the Board of Health Question, asked permission to report progress, promising to have a written report for December meeting. Moved and seconded that the report be accepted; carried.

Resolutions offered by Dr. Hanson and signed by Drs. O'Shea and Gill: That an amendment be made to Article IV. of the Constitution, in reference to annual election of officers, so as to correspond to change in Article XIII. of the By-laws, to read as follows: "Annual election shall take place on the evening of the first Wednesday in December." Moved and seconded that the resolution be accepted; carried.

Moved and seconded that the Secretary be authorized to purchase a rubber stamp, to be used to stamp on the face of application blanks that the initiation fee of five dollars must accompany the application for membership; carried.

Moved and seconded that the Secretary be authorized to notify members that election of officers will take place on the evening of the first Wednesday in December; carried.

Moved and seconded that the meeting adjourn; carried.

ROBERT W. ELLIS, D.V.S.,
Secretary.

